# EDTD-SC: An IoT Sensor Deployment Strategy for Smart Cities

**DOI:** 10.3390/s20247191

**Published:** 2020-12-15

**Authors:** Ibtihal Alablani, Mohammed Alenazi

**Affiliations:** 1 Department of Computer Engineering, CCIS, King Saud University, Riyadh 11451, Saudi Arabia; ealablani@tvtc.gov.sa; 2 Department of Information Technology, Technical College, Technical and Vocational Training Corporation, Riyadh 11451, Saudi Arabia

**Keywords:** smart city, sensor deployment, environmental monitoring, IoT, delaunay triangulation, coverage, end-to-end delay, network resilience, *k*-means, WSN

## Abstract

A smart city is a geographical area that uses modern technologies to facilitate the lives of its residents. Wireless sensor networks (WSNs) are important components of smart cities. Deploying IoT sensors in WSNs is a challenging aspect of network design. Sensor deployment is performed to achieve objectives like increasing coverage, strengthening connectivity, improving robustness, or increasing the lifetime of a given WSN. Therefore, a sensor deployment method must be carefully designed to achieve such objective functions without exceeding the available budget. This study introduces a novel deployment algorithm, called the Evaluated Delaunay Triangulation-based Deployment for Smart Cities (EDTD-SC), which targets not only sensor distribution, but also sink placement. Our algorithm utilizes Delaunay triangulation and *k*-means clustering to find optimal locations to improve coverage while maintaining connectivity and robustness with obstacles existence in sensing area. The EDTD-SC has been applied to real-world areas and cities, such as Midtown Manhattan in New York in the United States of America. The results show that the EDTD-SC outperforms random and regular deployments in terms of area coverage and end-to-end-delay by 29.6% and 29.7%, respectively. Further, it exhibits significant performance in terms of resilience to attacks.

## 1. Introduction

**Figure 1:**
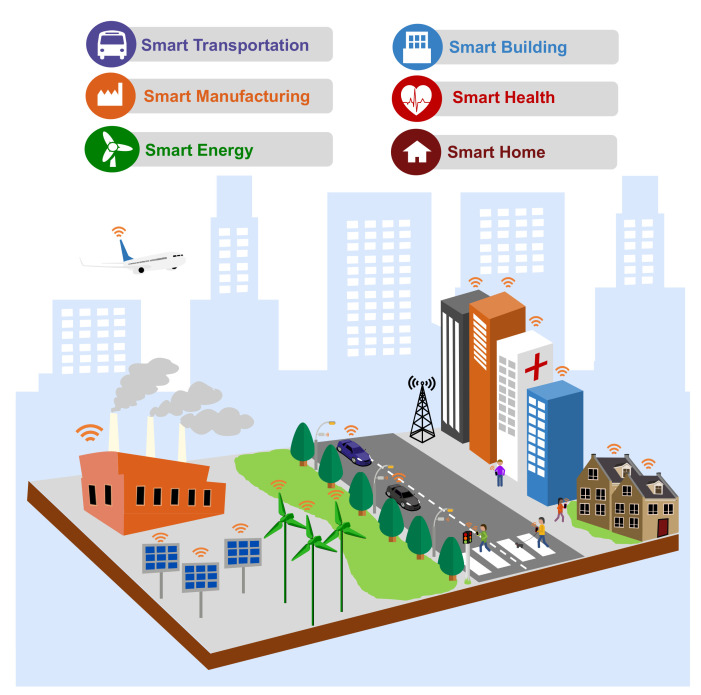
Typical smart city networked services.

A smart city is an urban environment where modern digital technologies are used to improve and facilitate the lives of its residents [1,2]. As shown in Figure 1, smart cities deal with critical and daily issues, including homes and buildings, environment, transportation, healthcare, energy, education, and manufacturing [3]. The Internet of Things (IoT) is an essential part of next-generation technology [4]. It is a promising concept that integrates a large number of objects, such as sensors, smartphones, actuators, houses, and appliances, into a network to use their data for real-time decision-making [5,6,7].

Environmental monitoring is an IoT application, where systematic sampling is performed to understand the nature of the environment (e.g., land, water, air, and biota) [8]. It can be applied in many areas, including natural disaster prevention, life-threatening situation avoidance, productivity improvement, and well-being and health enhancement [9]. Environmental issues where the IOT may be useful include the monitoring of air pollution, fires, agriculture, understory, hydrology, seismology, ecology, sunlight, habitat and microclimates [10,11]. Various environmental properties can be monitored using low-cost sensors [9].

Wireless sensor networks (WSNs) are a group of sensors organized to collect and process data about the phenomena of interest and then send them to a remote administrative center [4,12]. WSNs are critical components of smart cities, where the data collected by IoT sensors help in providing many services. WSNs have many applications in smart cities. These include smart buildings, traffic light control, parking optimization, intelligent transportation, energy management, structural health monitoring, waste management, surveillance, environmental monitoring, and pollution prevention [13,14].

Sensor deployment, which is the method of placing sensors in a desired area, is considered a challenging issue for researchers and developers. In WSNs, sensor deployment is a fundamental problem to be solved [15] because sensor deployment determines the coverage and connectivity of a WSN and its robustness against attacks [16]. In addition, it can prolong the lifetime of WSNs by reducing energy consumption [15]. The lifetime of a WSN refers to the time span from the deployment of a wireless sensor network to the time until the WSN has no ability to send useful information to the end-user [17]. It is mainly affected by the sensor’s battery capacity or solar sources. The appropriate sensor deployment strategy is chosen according to the application requirements and the cost of sensors [18]. Some examples of WSN application requirements are as follows.

- High coverage: Coverage is considered a main problem for almost all applications in WSNs. Multilevel (k) coverage of the sensing area is required by several applications [19]. Coverage is classified into full and partial coverage [20]. Some applications (e.g., surveillance application) require full coverage of a specific area [21]. However, full coverage is not necessary in some WSN applications [22].
- Low latency: Strict real-time applications (e.g., fire detection, earthquake, and intrusion detection) require a very low data transmission delay [23].
- High resilience to attacks: Some critical services, such as military-related applications, require a high degree of robustness against attacks because network resilience has an impact on its performance [24,25,26].
- Long lifetime: Some services require the WSN network lifetime to be long, as in underwater and harsh environment applications, where changing sensor batteries is a difficult task [27].

The contribution of this paper is the introduction of a new sensor/sink deployment strategy, called the Evaluated Delaunay Triangulation-based Deployment for Smart Cities (EDTD-SC). The proposed scheme has been designed for wireless sensor networks in smart cities and aims to distribute IoT sensors and sinks around the cities to improve the network performance. The sink distribution depends on a machine learning technique, called *k*-means clustering. The proposed algorithm considers the presence of obstacles in a region of interest. In WSNs, an obstacle is defined as a structure that prevents sensor placements or wireless communications. If a WSN is placed outdoors, buildings and mountains are considered to be obstacles. Furthermore, if a WSN is located indoors, walls, pillars, and fireplaces are examples of indoor obstacles. The EDTD-SC is applied to Midtown Manhattan with different scenarios to study the effect of varying numbers of obstacles, sinks, and IoT sensors on network performance. In addition, the EDTD-SC scheme is evaluated in terms of area coverage, end-to-end-delay, and resilience to attacks.

The remainder of this paper is organized as follows. Section 2 presents a brief background about the sensor deployment techniques and discusses related works. Section 3 represents preliminaries and the proposed sensor deployment strategy. Section 4 and Section 5 explain the experimental setup and results of and discussion on the EDTD-SC, respectively. Section 6 concludes the paper.

## 2. Background and Related Work

### 2.1. Background

Sensor deployment can be classified based on placement strategy into random and deterministic deployment [28].

#### 2.1.1. Random Deployment

**Figure 2:**
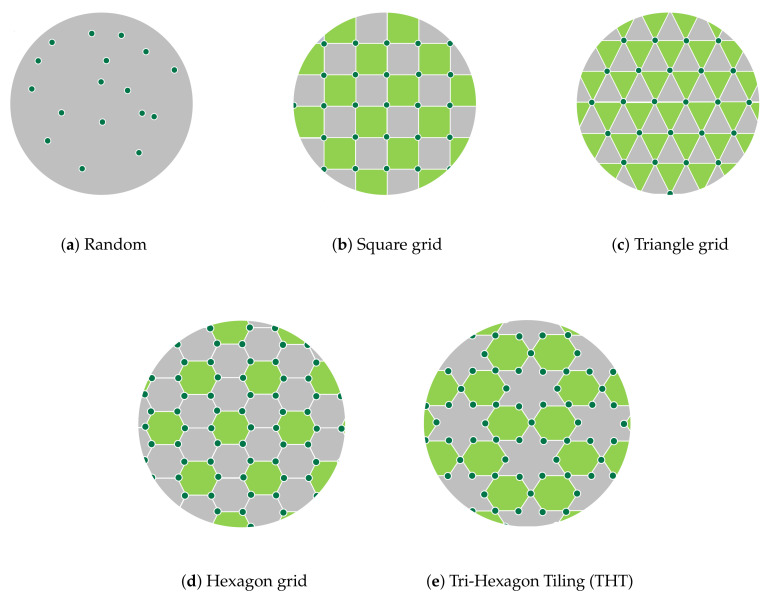
Examples of random and deterministic deployments.

In random deployment, sensors are randomly scattered over the region of interest to gather the target information. Figure 2a shows an example of random placement. It is suitable for regions where human existence is difficult (e.g., disaster areas, battlefields, air pollution, and forest fires) [29]. Random sensor deployment is preferred in many WSN applications due to the simplicity of the sensor distribution [15]. As a drawback, however, this method leads to uneven connectivity with critical sensors, which results in a network which is non-robust to sensor failure [30].

#### 2.1.2. Deterministic Deployment

In deterministic deployment, sensors are placed on the region of interest based on a certain geometrical structure [31]. Examples of this type of deployment are square, triangle, and hexagon grids, and tri-hexagon tiling (THT), as shown in Figure 2b–e, respectively [15].

A theoretical analysis in [32] proved that a hexagonal structure can provide a high coverage area with low energy consumption using a minimum number of sensors. Tri-hexagon tiling deployment was proposed to combine the advantages of the triangle and hexagon deployment methods. In terms of energy consumption, the THT deployment outperforms the square and hexagon deployments [33].

### 2.2. Related Work

This section presents previous work related to sensor deployment on wireless sensor networks. The work varies in terms of deploying objectives and strategies, based on the application of a wireless sensor network.

Chenxi et al. in [34] proposed the Distributed Voronoi-based Cooperation (DVOC) scheme. The DVOC scheme uses Voronoi diagrams and allows sensors to monitor the crucial points of other sensors around them by building local Voronoi diagrams (LVDs). Cooperation happens between sensors in detecting holes during movement. The experimental results show that DVOC outperforms other Voronoi-based schemes in terms of coverage and energy consumption. In [35], a node deployment method, called Voronoi-based Cooperative Node Deployment (VCOND), was proposed by Ghahroudi et al. The VCOND algorithm, based on the Voronoi diagram, uses neighborhood density and sensing coverage in an efficient manner to maximize the area coverage. The simulation result proved that the VCOND algorithm achieves a higher coverage percentage compared to other deployment methods and has less dependency on the initial node deployment.

Wu, M. proposed a sensor deployment strategy, called Neighbor node Location-based Coverage Hole Recovery in [36], which restores coverage holes by deploying mobile sensors at an optimal position through an exchange of information with the neighboring boundary sensors. It is simple to apply, compared to other deployment schemes based on the Voronoi diagram or Delaunay triangulation, to restore coverage holes. In [37], Khedr et al. proposed a WSN scheme, called Coverage Aware Planar Distributed Topological Face Structure (CAFT). Based on the CAFT strategy, a number of sensors are organized to achieve effective coverage and connectivity, while the remaining sensors remain in sleep mode to decrease energy consumption and prolong the WSN lifetime. This scheme has two main phases: the topology construction and target detection and tracking phases. The simulation results showed that the CAFT approach can reduce energy consumption and enhance the WSN lifetime.

Meanwhile, Yarinezhad, R. and Hashemi, S.N. improved the particle swarm optimization (PSO) algorithm by proposing two sensor deployment methods: cooperative PSO (CPSO) and cooperative PSO, using fuzzy logic (CPSO-Fuzzy). Their simulation results demonstrated that the proposed approaches can solve the target coverage problem. Furthermore, their network lifetimes were longer than other existing methods [38]. In [39], Arivudainambi and Pavithra developed a sensor deployment scheme based on vertex coloring. This scheme was designed for applications with three-dimensional terrains. The breadth-first search algorithm was used to determine the connectivity quality of sensors. The results showed that the proposed scheme has more efficient coverage and connectivity than other similar algorithms. In [40], Zhaoyong proposed a node deployment scheme for heterogeneous wireless sensor networks. The deployment model was designed based on the prediction of the possible consumption of traffic and energy. The simulation results demonstrated that using the improved technique enhances the coverage percentage and energy consumption compared to other existing methods. Furthermore, the complexity and the overhead are restricted within a lower value.

The limitations are summarized as follows, based on the work presented in this section.

- The focus is on deploying sensor nodes in a WSN. The aspect of sink distribution is neglected.
- The existence of obstacles in a sensing area is not considered when designing a sensor deployment algorithm.

## 3. Proposed Deployment Strategy

### 3.1. Preliminaries

In the preliminary section, the Voronoi diagram, Delaunay triangulation, and *k*-means clustering algorithm are introduced to convey a good understanding of our EDTD-SC approach.

#### 3.1.1. Voronoi Diagram

**Figure 3:**
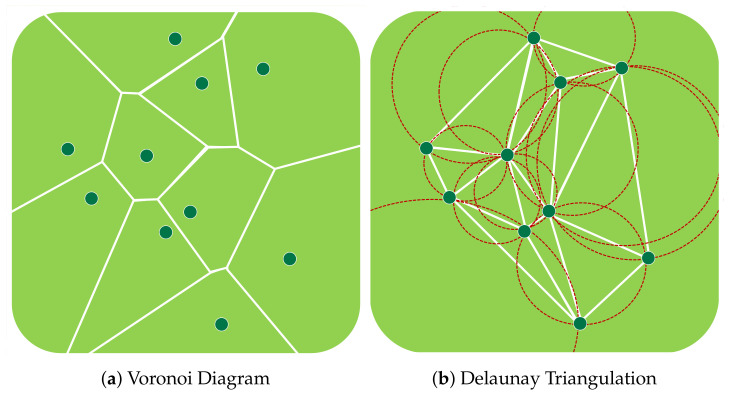
Voronoi diagram and Delaunay triangulation.

The Voronoi diagram was proposed by Rene Descartes in 1644. For a set of random points, P, a Voronoi diagram VD(P) is set in Voronoi regions, called Voronoi cells (i.e., one for each point in P) [41]. The locations inside a Voronoi cell are closer to their corresponding point than the other points [42] (Figure 3a).

#### 3.1.2. Delaunay Triangulation

Delaunay triangulation (DT) is a triangular mesh that links a group of points in a plane [43]. DT was proposed by Boris Delaunay in 1934. For a set of random points, P, the Delaunay triangulation DT(P) is dual to its Voronoi diagram (Figure 3b). The Delaunay triangulation of P is a triangulation such that no point in P exists inside the circumcircle of any DT(P) triangle [41].

#### 3.1.3. *k*-Means Clustering Algorithm

**Figure 4:**
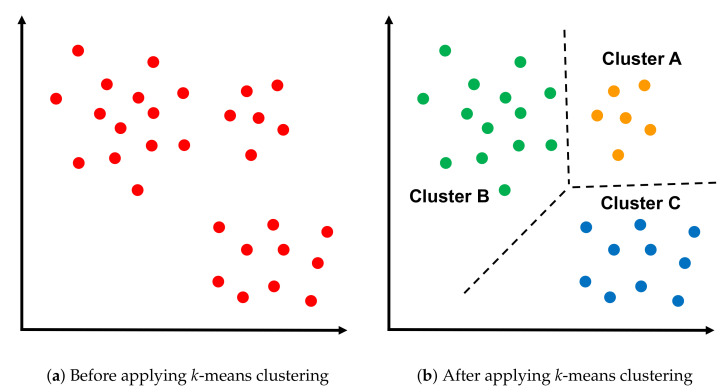
Applying *k*-means clustering algorithm on set of points.

The *k*-means clustering algorithm is an unsupervised machine learning algorithm that aims to divide n observations into clusters (Figure 4). An observation is assigned to the cluster with the closest mean. The *k*-means algorithm has three stages: initialization, computation, and convergence (Algorithm 1) [44].
**Algorithm 1:** Pseudocode for *k*-means clustering
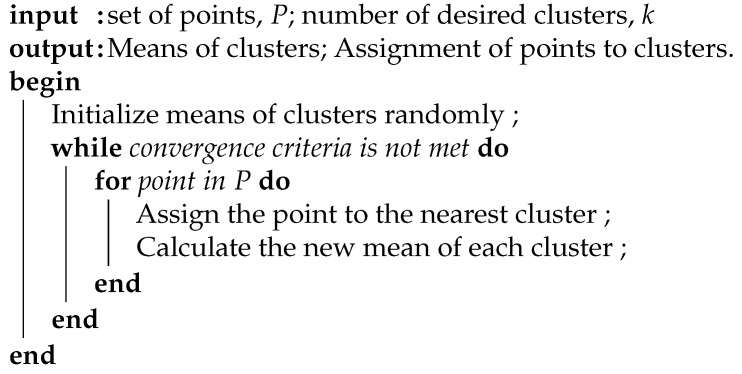


### 3.2. EDTD-SC Algorithm

This section explains the proposed sensor deployment scheme (i.e., EDTD-SC) in detail. The EDTD-SC algorithm was designed for IoT smart cities with obstacles. It focuses on determining the sensor locations and identifying the suitable sink positions based on the *k*-means clustering algorithm, because a proper selection of sink locations positively affects the WSN performance. Accordingly, the EDTD-SC strategy consists of three main phases: configuration, sensor deployment, and sink deployment.

#### 3.2.1. Configuration Phase

**Figure 5:**
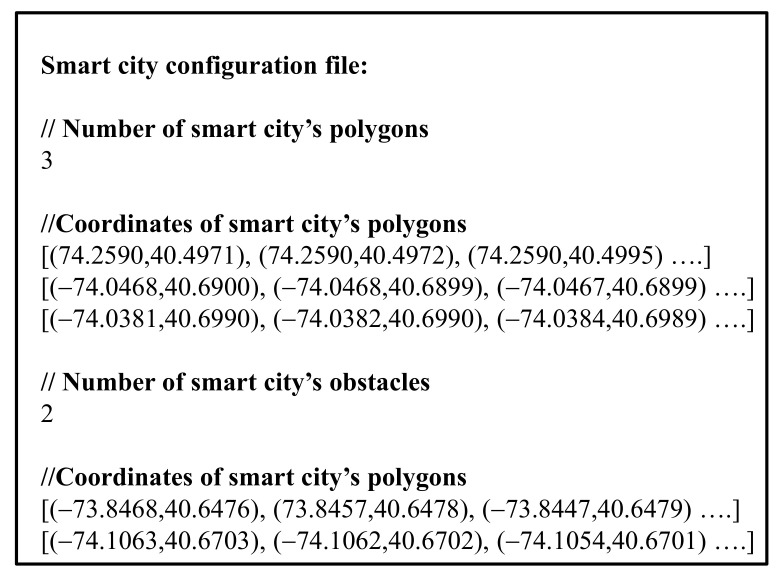
Example of a smart city configuration file.

A smart city configuration file is read in the configuration phase. This file contains information about the polygons that make up a smart city map and the obstacles within the city. Figure 5 shows an example of a smart city configuration file.

#### 3.2.2. Sensors Deployment Phase

**Figure 6:**
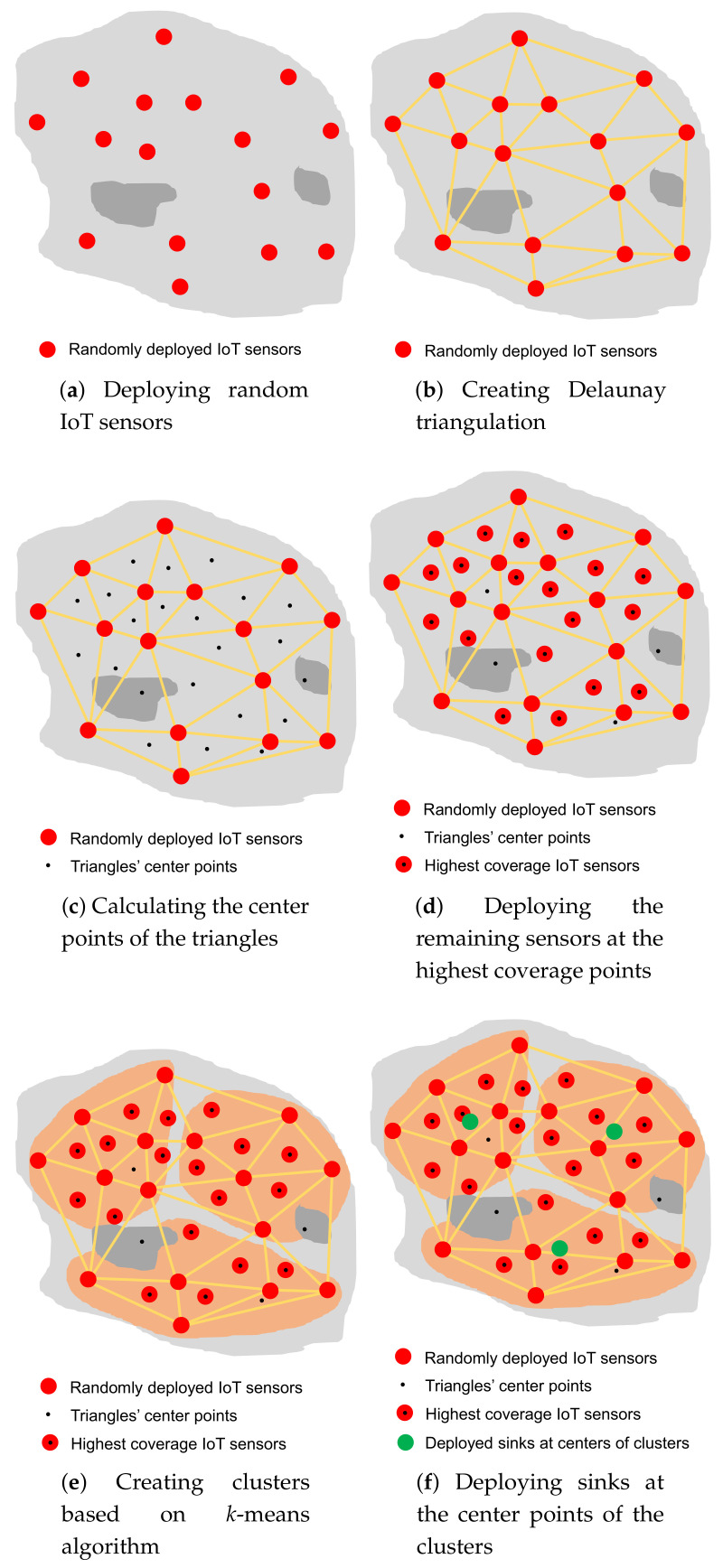
Illustration of the EDTD-SC deployment strategy.

In this phase, IoT sensors are deployed throughout a smart city. The sensor deployment phase includes the three following steps:

1. Random Location Generation: In this step, a set of random places is generated based on the assumed percentage of random points (i.e., 50%). Subsequently, IoT sensors are placed on random points, provided that they are within the boundaries of a smart city and not within any obstacle. Figure 6a illustrates the random location generation step in a simple example.
2. Delaunay Triangulation Creation: In this step, depending on the locations of the sensors deployed in the previous step, triangles are created based on Delaunay triangulation (Figure 6b). A center point is then calculated for each triangle (Figure 6c).
3. Coverage Evaluation Step: In this step, the triangle center points are evaluated based on the coverage percentage. Sensors will be deployed in the center points with the highest coverage ratio, depending on the available number of IoT sensors (Figure 6d). Therefore, deploying sensors in areas that have a small number of sensors (including areas around obstacles) has a higher priority than other points.

#### 3.2.3. Sinks Deployment Phase

This phase aims to deploy sinks to be close to all sensor nodes to achieve a high WSN performance. To achieve this goal, the *k*-means clustering algorithm is applied to sensors deployed in the previous phase. The sensors are divided into clusters (i.e., three clusters in this example) equal to the desired number of sinks (Figure 6e). The sinks are then placed on the center points of the clusters (Figure 6f). If the center point is located on an obstacle, it will be moved to the nearest point on the obstacle boundary. In this way, our EDTD-SC algorithm ensures that areas around obstacles are covered and there are no disconnected sensors. Algorithm 2 shows the pseudocode of the EDTD-SC algorithm. The code illustrates the three main phases of the EDTD-SC deployment scheme.
**Algorithm 2:** Pseudocode for EDTD-SC
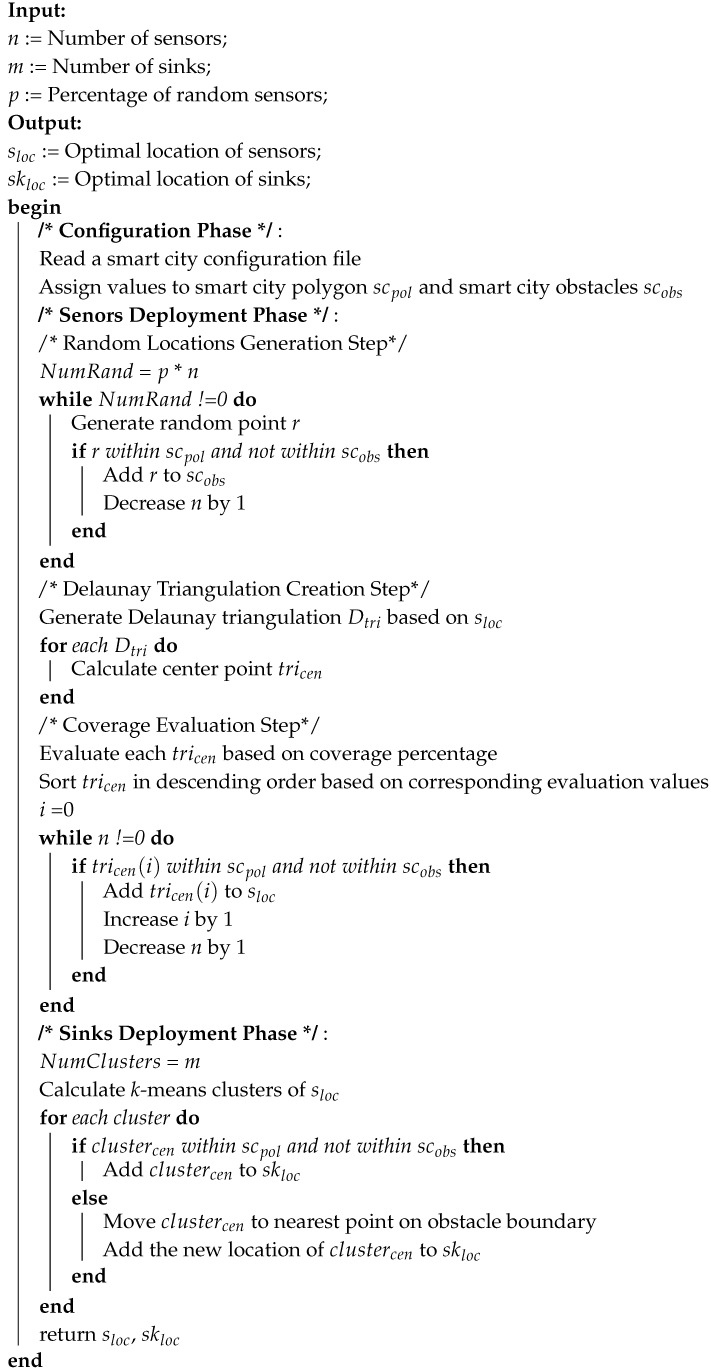


## 4. Experimental Setup

This section describes the experimental tools, functions and assumptions that were considered to implement and evaluate the proposed scheme. Python programming language was utilized for this, due to its powerful capabilities [45]. The Shapely package, a Python package that eases work with polygons [46], was employed to create polygons in the smart city and perform geometric operations. NetworkX, a Python package that facilitates working on graphs and networks [47], was utilized to create a WSN and perform networking operations. OpenStreetMap, which is a free world map [48], was used to obtain a map of the polygons in the smart city considered in this work and the locations of obstacles. A projection library was used to convert geographic coordinates (longitude and latitude) to projected coordinates. It was assumed that the percentage of random sensors used in EDTD-SC strategy is 50%.

## 5. Results and Discussion

**Figure 7:**
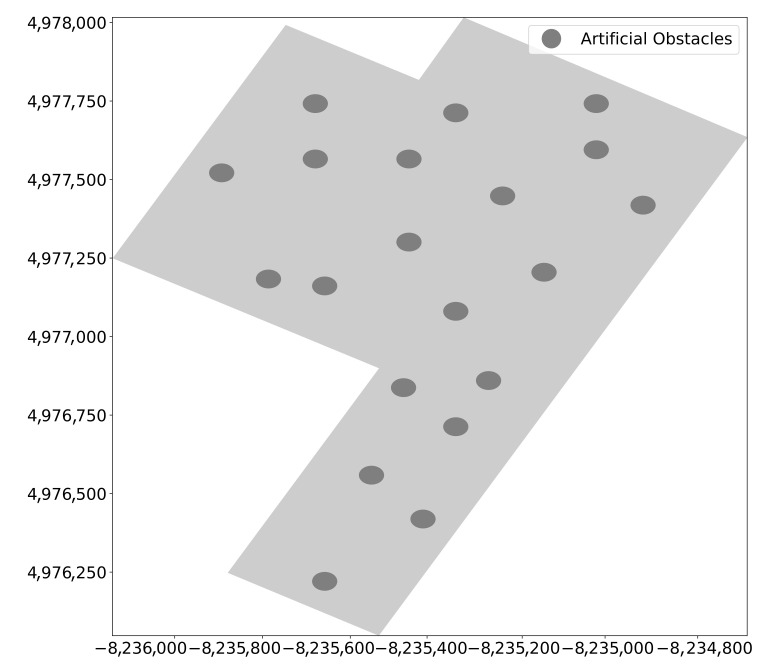
Midtown Manhattan map.

**Figure 8:**
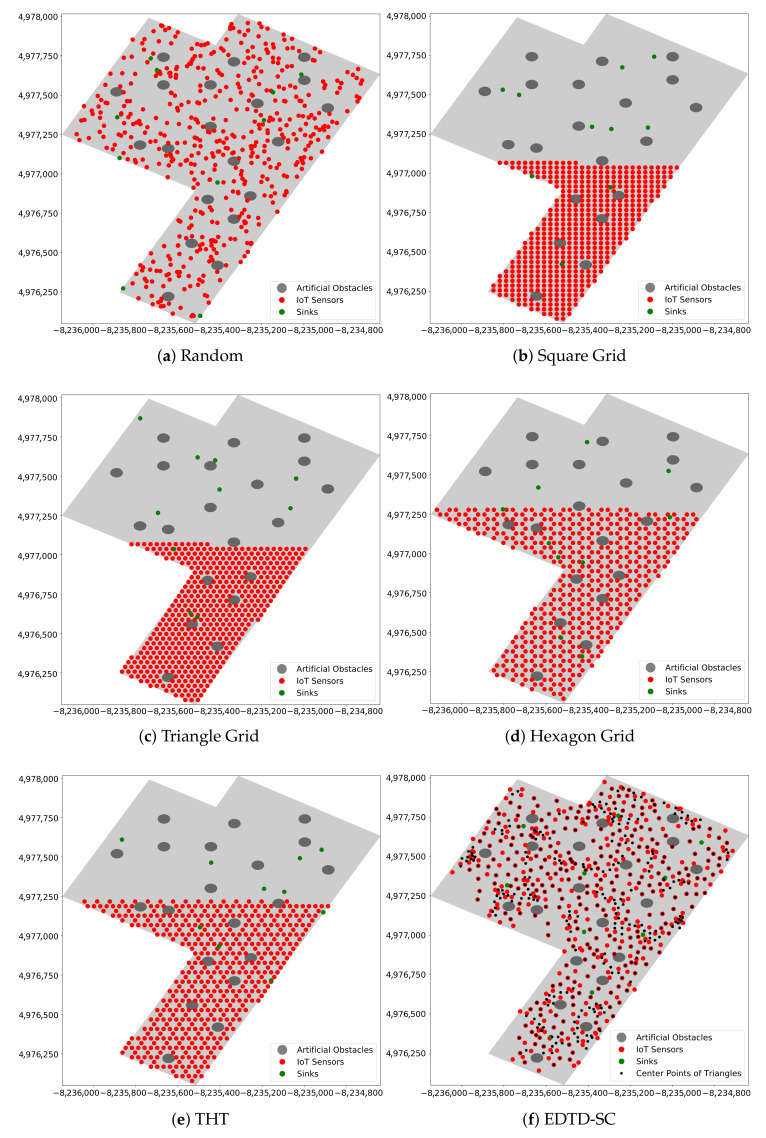
Applying sensor deployment strategies on Midtown Manhattan.

The performance of the EDTD-SC was evaluated in terms of area coverage, end-to-end-delay, and resilience to attacks. The EDTD-SC was applied to Midtown Manhattan in New York in the US. When implementing the experiment, twenty artificial obstacles were placed randomly in the sensing area. Figure 7 shows the map of Midtown Manhattan, which is considered a relatively small area, which includes the locations of obstacles. Five deployment schemes were considered: a random, a square grid, a triangle grid, a hexagon grid, and THT. Figure 8 illustrates the deployment of 500 sensors and 10 sinks on Midtown Manhattan using different deployment strategies.

### 5.1. End-To-End-Delay

The end-to-end delay, which is the time required for a packet to be transmitted across a WSN from a sensor to a sink, was calculated based on Equation (1). The end-to-end, processing, queuing, transmission, and propagation delays are denoted herein by $d_{e2e}$, $d_{proc}$, $d_{queu}$, $d_{tran}$, and $d_{prop}$, respectively. Processing delay is defined as the time required for computing and processing packets by the sensors [49]. It is based on the computational capabilities of sensors in the WSN [50]. In this study, it is assumed that all sensors have high processing capabilities. Thus, the processing delay is insignificantly small.

(1) $$d_{e2e}=d_{proc}+d_{queu}+d_{tran}+d_{prop}$$

The transmission delay is a packet of size L bits divided by the transmission rate R in bps (Equation (2)). ZigBee, IEEE 802.15.4, is the most widespread technology used for WSNs [51]. It is the current de facto standard, as almost all existing commercial and research sensors are operated using ZigBee transceivers [52]. It has been proven that ZigBee is one of the best candidates for WSN design due to its excellent performance on connectivity and power consumption [53]. L was assumed as the maximum package size of ZigBee networks, which is 128 bytes [54]. ZigBee data rates range from 20 to 250 kbps [55]. In this paper, R was assumed to be 250 kbps, which is the maximum data rate supported by ZigBee technology.

(2) $$d_{tran}=\frac{L}{R}$$

The propagation delay is the distance between a sensor and a sink D (in meters) divided by velocity V (in m/s), as represented in Equation (3). The actual distance between a sensor and its nearest sink is calculated according to Python’s NetworkX package. We assumed V to be the velocity of light, equal to 300 m/µs.

(3) $$d_{prop}=\frac{D}{V}$$

**Figure 9:**
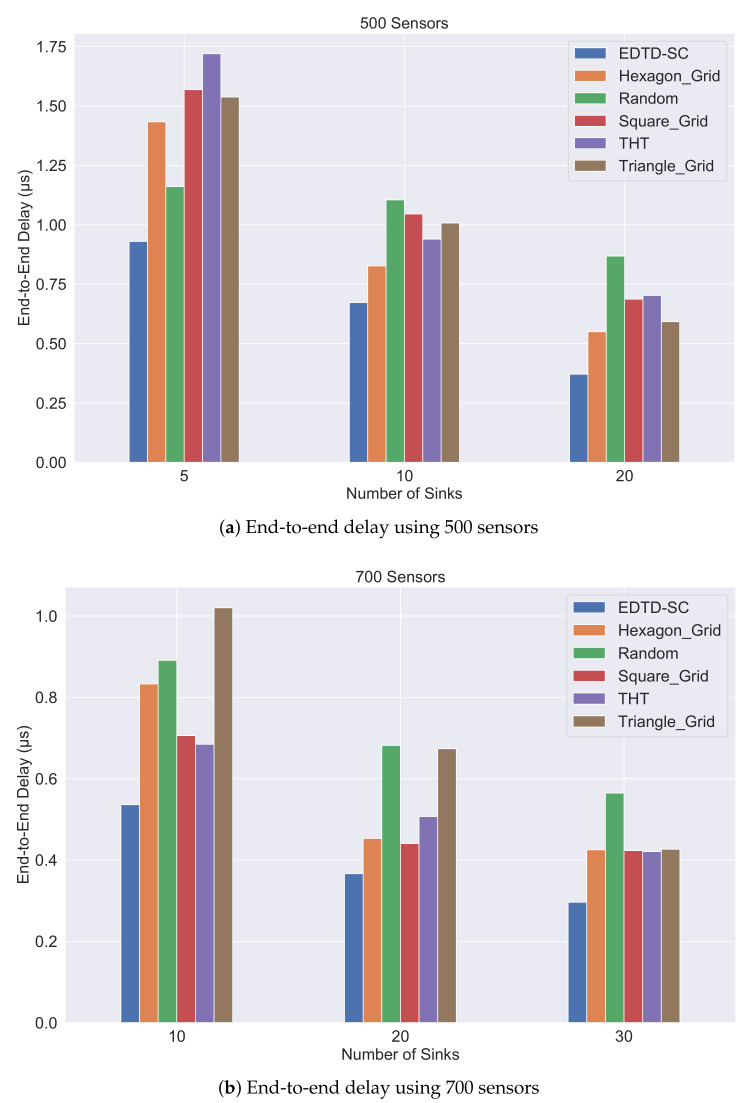
End-to-end delay comparison of six strategies in different scenarios.

The results of deploying 500 sensors with different numbers of sinks are shown in Figure 9a. The results show that the EDTD-SC algorithm consistently provides the minimum end-to-end delay of 0.92 μs, 0.66 μs, and 0.37 μs for 5, 10, and 20 sinks, respectively. For all methods, we notice that as the number of sinks increases, the end-to-end delay decreases. This is because a sink’s location close to the sensors will reduce propagation delay and the number of hops. In the random deployment method, the decrease in delay with the increase in the number of sinks occurs slowly compared to the regular deployment strategies. Adding 15 extra sinks leads to a reduction in delay by 25% in random deployment and by 56% and 59% for square grid and THT, respectively. The remaining methods, including EDTD-SC, achieve 60% reduction in delay with increasing numbers of sinks. The reason for slow interaction in random deployment is the variation in distances between the 500 sensors in a sensing area, which were randomly deployed without relying on a specific methodology.

The results of deploying 700 sensors with different numbers of sinks are shown in Figure 9b. The results show that the EDTD-SC algorithm also provides the minimum end-to-end delay with values 0.52 μs, 0.36 μs, and 0.29 μs in cases of 10, 20, and 30 sinks, respectively. Based on the bar chart, adding 20 extra sinks leads to decreasing the delay by 37% in random deployment, which is considered the lowest decreasing percentage compared to the other sensor deployment strategies.

We can say that EDTD-SC is the best in terms of end-to-end delay compared to its competitors. The first reason is that, in terms of sink location, EDTD-SC relies on the *k*-means machine learning technique. Therefore, sinks will be close to the sensors, and the end-to-end delay will be reduced. The second reason is that the EDTD-SC scheme relies on reducing those areas that were not covered in the first step of the sensor deployment phase (random location generation step). This is achieved in the second and third steps by distributing sensors at the center points of Delaunay triangulation that have the highest coverage ratio. In this way, distances between the sensors decrease, and therefore the end-to-end delay is reduced.

### 5.2. Area Coverage

In WSNs, the area coverage metric measures how closely a sensing area is monitored by the sensors. The coverage percentage can be calculated by dividing the covered area by the total target area (Equation (4)),

(4) $$Coverage\left(\%\right)=\left(\frac{A_{covered}}{A_{total}}\right)\times 100$$

where $A_{covered}$ is the geographical area covered by the sensors and $A_{total}$ is the total geographical area of the target region to be covered. We assume that areas inside obstacles should not be covered. Therefore, these areas are subtracted from the coverage area.

**Figure 10:**
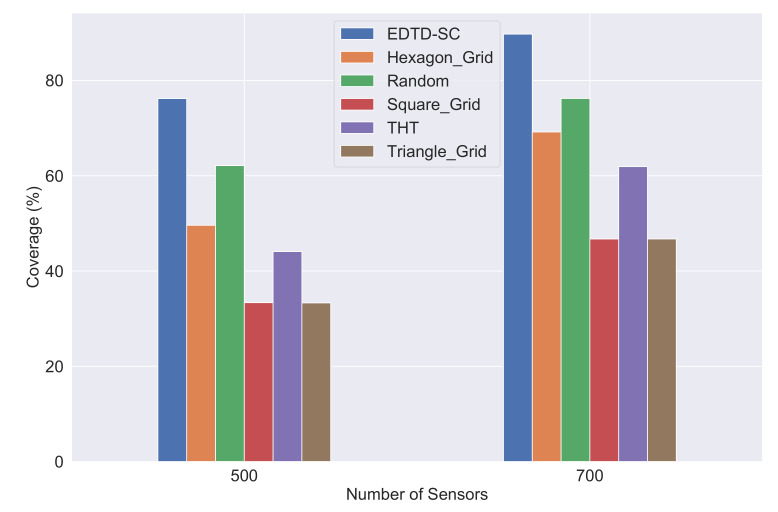
Area coverage comparison of the six strategies in different scenarios.

Figure 10 depicts the analytical results of the area coverage comparison among the six strategies, using different numbers of sensors. The result illustrates that the proposed EDTD-SC deployment algorithm consistently provides the greatest coverage percentage of 73.75% and 86.84% in cases of 500 and 700 sensors, respectively. Random deployment yields the second highest value of coverage percentage with 16.76% lower coverage, on average, compared to the EDTD-SC algorithm. The hexagon grid and THT grid deployment are ranked as the third and the fourth deployment methods, respectively, in terms of area coverage with percentages 28.9% and 36.56% lower, on average, compared to the EDTD-SC algorithm. The reason for this is that the hexagon shape is characterized by variations in the internal distances between its points. This is followed by the hexagonal star shape, as shown in Figure 8d,e.

As a result, the EDTD-SC deployment strategy outperformed its competitors in all scenarios, because it is based on Delaunay triangulation to cover holes after deploying random sensors in a sensing area. In addition, it evaluates the center points of Delaunay triangulation and places sensors in high coverage points.

### 5.3. Resilience to Attacks

Network resilience is an important concept that should be considered in WSN design [56]. It is the ability of a network to overcome changes that may happen, for example, in network topology and link configuration [57]. Two kinds of network attacks were considered in this study: degree- and betweenness-based sensor attacks. The former means attacking sensors with the highest number of edges (degree), while the latter means attacking sensors with the largest number of shortest paths crossing through them (betweenness).

**Figure 11:**
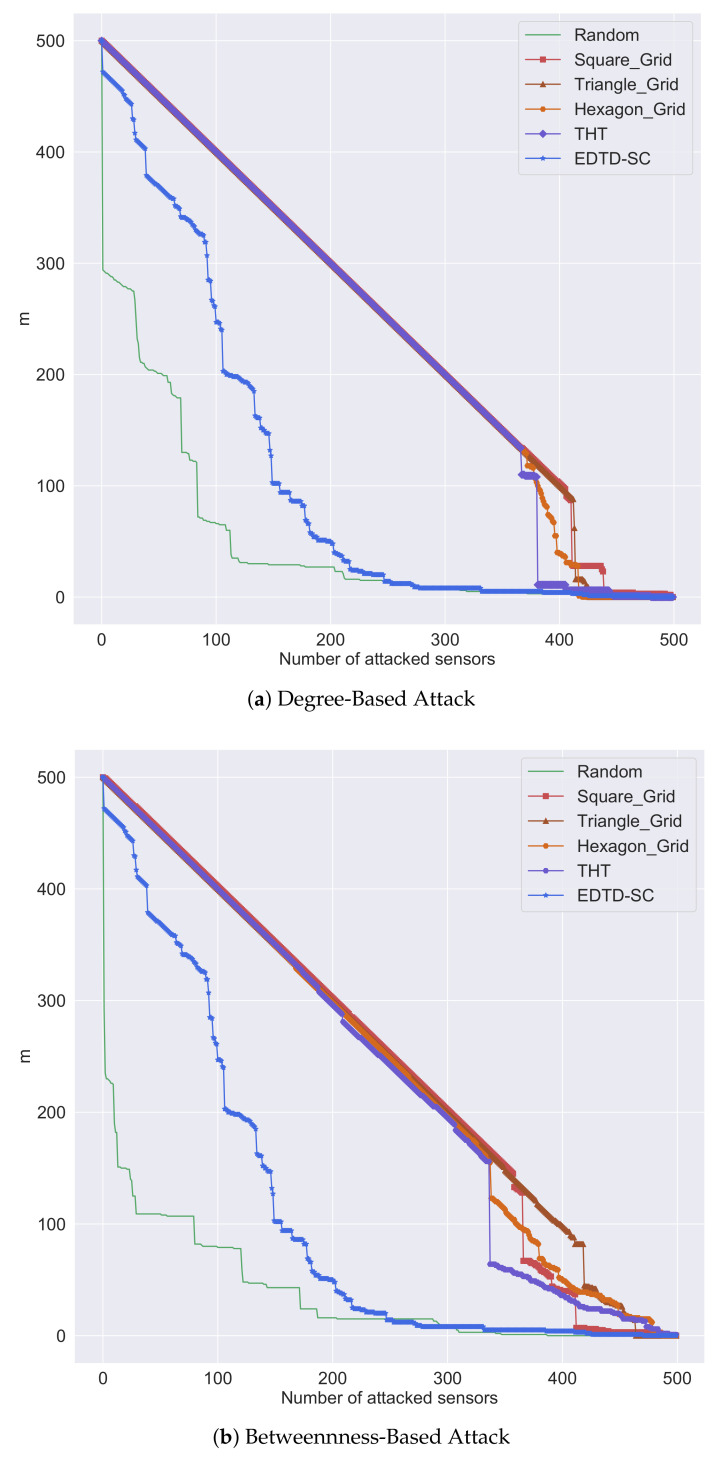
Result of resilience to attacks using 500 sensors and 10 sinks.

Figure 11a,b show the impact of degree- and betweenness-based sensor attacks, respectively, on network resilience. The line charts represent the relationship between the number of sensors attacked and the number of sensors connected to any sink (m) at the time of attack. The random sensor deployment scheme is the worst in terms of resilience against attacks because consistency between the sensors is not considered during the sensor deployment phase. In contrast, the regular sensor deployment strategies are the best in terms of resilience to attacks because a high cohesion exists between the sensors. The EDTD-SC algorithm has an acceptable performance in the face of attacks, remaining intact and not collapsing rapidly as in a random distribution.

As shown in this section, the EDTD-SC deployment algorithm outperformed its five competitors. In terms of end-to-end delay and area coverage, the EDTD-SC strategy outperformed other methods by an average percentage of 29.7% for delay and 29.6% for coverage. In addition, it has an adequate performance in terms of resilience to attacks. Based on the results, the EDTD-SC algorithm is suitable for the placement of sensors and sinks in environmental monitoring applications, such as measuring air pollution, weather temperature, humidity, and rainfall. Moreover, our algorithm can provide better connectivity and sensing during natural disasters, such as earthquakes and hurricanes.

### 5.4. Impact of Increasing Number of Obstacles

**Figure 12:**
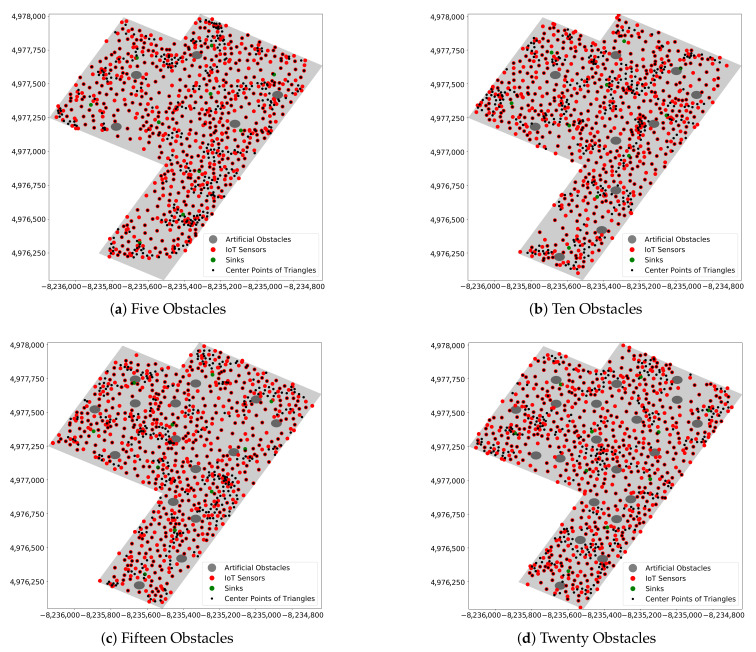
Applying EDTD-SC deployment scheme on Midtown Manhattan using different numbers of artificial obstacles.

**Figure 13:**
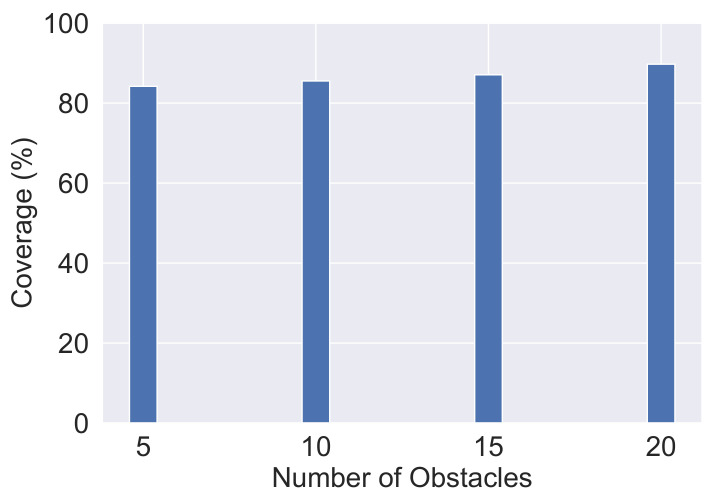
Impact of obstacles existence on coverage percentage.

The effect of the existence of artificial obstacles in Midtown Manhattan on network performance is presented in this section. Figure 12 shows the application of the EDTD-SC deployment strategy to Midtown Manhattan with 700 deployed IoT sensors and ten sinks. In addition, different numbers of artificial obstacles are assumed: five, ten, fifteen, and twenty obstacles. The coverage percentage is evaluated to study the impact of the presence of obstacles. Figure 13 illustrates that the coverage area increases by an average of 2.14% with an increase in the number of obstacles. The reason is that, based on the EDTD-SC strategy, IoT sensors should not be placed on the obstacles. Thus, the area of obstacles is subtracted from the total area of the sensing region. Therefore, the presence of obstacles in the sensing area leads to a reduction in the area to be covered, based on the area of the obstacles.

## 6. Conclusions and Future Work

Sensor deployment in WSNs is an important issue that needs to be addressed. This study proposed a three-phase deployment strategy, called EDTD-SC, for WSNs in smart cities. The EDTD-SC is a deployment approach suitable for determining the locations of sensors and sinks in a sensing area with obstacles. The first phase involves reading a configuration file including information about the polygons that make up a smart city map and the obstacles within the city. The second phase involves sensor deployment, comprising three steps: random location generation, Delaunay triangulation creation, and coverage evaluation. The last phase is sink distribution, based on the *k*-means clustering machine learning technique. As a result, the proposed scheme outperformed the random and four regular deployment strategies in terms of the end-to-end delay and the area coverage, which are the most important performance metrics in WSN applications. In terms of end-to-end delay, the EDTD-SC algorithm achieves improvement over its competitors with an average percentage of 29.7%. In addition, the proposed strategy has a superiority, on average, of 29.6% in terms of area coverage. Furthermore, the EDTD-SC algorithm has an acceptable performance in terms of resilience against degree-based and betweenness-based attacks. For future work, the EDTD-SC algorithm can be compared with different strategies, such as DVOC, VCOND, CAFT, CPSO, and CPSO-Fuzzy. Furthermore, the EDTD-SC can be applied to other cities, such as Florida City, Cleveland, New York, and Los Angeles City to study the impact of varying geographical sizes on network performance.
